# Purinergic neurotransmission receptor P2X4 silencing alleviates intracerebral hemorrhage-induced neuroinflammation by blocking the NLRP1/Caspase-1 pathway

**DOI:** 10.1038/s41598-023-40748-8

**Published:** 2023-08-31

**Authors:** Yuanshui Wu, Xiaoli Huang, Le Yang, Yuanjie Liu

**Affiliations:** 1 Department of Neurosurgery, ShangRao People’s Hospital, No. 87, Shuyuan Road, Shangrao City, 334000 Jiangxi Province China; 2 JiangXi Medical College, No. 399, Zhimin Road, Xinzhou District, Shangrao City, 334099 Jiangxi Province China; 3 Department of Neurosurgery, Nanfang Hospital, Southern Medical University, No. 1838, Guangzhou Avenue North, Guangzhou City, 510515 Guangdong Province China

**Keywords:** Biological techniques, Cell biology, Molecular biology

## Abstract

This study is performed to explore the role of P2X4 in intracerebral hemorrhage (ICH) and the association between P2X4 and the NLRP1/Caspase-1 pathway. The mouse ICH model was established via collagenase injection into the right basal ganglia. P2X4 expression in brain tissues was knocked down via intracerebroventricular injection with adeno-associated virus (AAV) harboring shRNA against shP2X4. The gene expression of P2X4 and protein levels related to NLRP1 inflammasome were detected using qRT-PCR and Western blot analysis, respectively. Muramyl dipeptide (an activator of NLRP1) was used to activate NLRP1 in brain tissues. ICH induced high expression of P2X4 in mouse brain tissues. The knockdown of P2X4 alleviated short- and long-term neurological deficits of ICH mice, as well as inhibited the tissue expression and serum levels of pro-inflammatory cytokines, including TNF-α, interleukin (IL)-6, and IL-1β. Additionally, the expressions of NLRP1, ASC, and pro-Caspase-1 were down-regulated upon P2X4 silencing. Moreover, neurological impairment and the expression and secretion of cytokines after P2X4 silencing were aggravated by the additional administration of MDP. P2X4 knockdown represses neuroinflammation in brain tissues after ICH. Mechanistically, P2X4 inhibition exerts a neuroprotective effect in ICH by blocking the NLRP1/Caspase-1 pathway.

## Introduction

Intracerebral hemorrhage (ICH) is one of the typical stroke syndromes, which accounts for about 15% of all strokes, and there are approximately 2.8 million die of this disease worldwide each year^1,2^. According to statistics, lower-income countries have much higher morbidity and mortality of ICH than high-income countries^3^. Moreover, globally, the incidence of ICH among Asians is higher than that among most other racial groups^4^. ICH includes spontaneous and nontraumatic ICH and secondary ICH^5^. There is a series of causes that can lead to ICH, such as the cerebral microvasculopathy associated with chronic hypertension and cerebral amyloid angiopathy (CAA)^6,7^. Besides, a minority of ICH results from some “macrovascular” bleeding sources including arteriovenous malformations, cavernomas, or fistulas^8^. However, there have been few breakthroughs in outcomes after ICH and definitive management of ICH^5,9^.

P2X receptors, the receptors for purinergic neurotransmission, are a family of non-selective trimeric ligand-gated channels. Upon combination with extracellular ATP, they permit Na^+^, K^+^, and Ca^2+^ ion fluxes^10^. P2X4, a typical P2X receptor, is one of the most sensitive purinergic receptors^11^. P2X4 is expressed in most cells in the central and peripheral neurons, microglia, and various glandular tissues^12^. P2X4 receptor is generally overexpressed in most neurological conditions^13^. P2X4 has been reported to be up-regulated in spinal cord injury, neuropathic and inflammatory pain, ischemia, and other disease models which involve microglial activation^14,15^. Blockade of P2X4 signaling exacerbates clinical signs in the experimental autoimmune encephalomyelitis (EAE) model and also favors microglia activation to a pro-inflammatory phenotype^16^. The acute purinergic receptor P2X4 blockade exerts neuroprotective and neuro-rehabilitative effects after ischemic stroke by reducing the numbers of infiltrating pro-inflammatory myeloid cells^17^. These previous findings reveal the vital role of P2X4 in neuroinflammation.

Inflammasomes are intracellular multi-protein complexes. lipopolysaccharide (LPS), oxidative stress, potassium efflux, and other factors can activate inflammasomes which trigger the maturation and expression of proinflammatory cytokines to initiate innate immune responses^18,19^. Nucleotide-binding domain, leucine-rich repeat (NLR) protein 1 (NLRP1) and NLRP3 inflammasomes are the most thoroughly studied. Knockdown of P2X4 receptor expression has been demonstrated to restrict the activation of the NLRP3 inflammasome and alleviates dopaminergic neurodegeneration and neuroinflammation^20^. Chinese herbal compound Naofucong is demonstrated to be able to ameliorate hippocampal neuron injury induced by high glucose via blockade of P2X7/NLRP1/Caspase-1 pathway^21^, which indicates a crucial role of NLRP1 in neuron injury. However, the role of P2X4 in NLRP1 inflammasome is still unknown.

The effect of P2X4 on neuroinflammation upon ICH and its association with NLRP1 are still mysterious. As mentioned above, P2X7 can mitigate neuron injury via the NLRP1/Caspase-1 pathway. Besides, there is an interaction between P2X4 and P2X7^22^. We wonder whether P2X4 can regulate neuroinflammation after ICH via the NLRP1/Caspase-1 pathway. In this study, we established a mouse ICH model and knocked down P2X4 in mice to confirm our speculation.

## Materials and methods

### Mouse ICH model

The 8-week-old male CD1 mice (Charles River, Wilmington, USA) weighing 30–32 g were used for the establishment of the ICH model. In the brain tissue surrounding the hematoma in the right basal ganglia region, collagenase was injected into the right basal ganglia to induce ICH. Then, all mice were anesthetized with ketamine (100 mg/kg) and dexmedetomidine (0.5 mg/kg) via intraperitoneal injection. Subsequently, the mice were immobilized on a brain stereotaxic apparatus (RWD, China). A 1-mm crenocloma was drilled, and a 26-gauge needle was inserted stereotactically and 0.075 units of collagenase (type VII-S; Sigma-Aldrich, 9001-12-1, St. Louis, MO, USA) in 0.5 μl sterile phosphate-buffered saline (PBS) was injected into the right basal ganglia. Then the needle was kept at the injection point for 5 min in case of liquid backflow. After the cranial pinhole was blocked with bone wax, and the skin was sutured, 0.4 ml of saline was injected subcutaneously. In sham group, the needles were inserted with equivalent PBS, and the other operations were identical. All animal experiments were approved by the Institutional Animal Care and Use Committee of ShangRao People’s Hospital.

### Animal grouping and administration

To detect the expression of P2X4 in ICH model, the mice were randomly divided into five groups: Sham, 6 h, 24 h, 72 h, 7d, and 28d. Mice were euthanized 6 h, 24 h, 72 h, 7d, or 28d, post-ICH, and ipsilateral hemispheres of all mice were removed for expression detection.

To assess the effect of P2X4 knockdown, the mice were divided into five groups: Sham, ICH, AAV-shNC, AAV-shP2X4-1, and AAV-shP2X4-2. The mice in AAV-shP2X4-1 group and AAV-shP2X4-2 group were injected with adeno-associated virus (AAV; Thermo Fisher Scientific, A47672, Massachusetts, USA) harboring two different shRNAs against P2X4 (AAV-shP2X4), respectively, via intracerebroventricular administration 24 h before ICH induction. AVV containing scrambled shRNA served as a negative control (AAV-shNC).

To explore the association of P2X4 with NLRP1, muramyl dipeptide (MDP; Sigma-Aldrich, A9519, Missouri, USA), an activator of NLRP1 was injected into the mice at a dose of 5 mg/kg.

### Assessment of neurological deficits

Neurological functions including the modified Garcia test, corner turn test, and limb placement were evaluated by a researcher blinded to the experimental design. These tests were carried out 24 h after ICH to assess short-term neurological deficits^23^. The modified Garcia test includes the evaluations of spontaneous activity, axial sensation, vibrissae proprioception, limb symmetry, lateral turning, forelimb walking, and climbing. In the corner turn test, mice were allowed to approach a 30° corner. When the mice left the corner, they either turned right or left. The tests were performed 10 times with at least a 30-s rest period between each trial. The forelimb placement test was assessed by recording the placement of the ipsilateral forelimb on the countertop upon vibrissa stimulation. The percent of the ipsilateral forelimb placement over ten total trials was calculated.

On day 28 after the occurrence of ICH, the Morris water maze test and rotarod test were performed. Morris’s water maze was used to test the recovery of spatial learning ability and memory function based on previous studies for the evaluation of long-term neurological deficits^24^. Each mouse started at a semi-random location and then was allowed to search the partially submerged platform for 60 s. Next, they were led to the platform, where they stayed for 5 s. On the final day, the probe trial was conducted. The platform was removed upon the learning trial. A camera connected to a computer tracking system (Noldus Ethovision, WA, USA) was used to record swimming path, frequency of platform crossings, the latency of first platform crossing, and frequency of correct quadrant crossings of the mice. In the rotarod test, the rotational speed of the cylinder was slowly elevated from 4 to 40 rpm within 5 min. The mice run on the cylinders until they fell off, and the times were recorded. Each mouse underwent tests three times, and the average retention time of rotarod was calculated.

### AAV-shRNA-P2X4 construction and AAV virus packaging

The AAV-shRNA-P2X4 plasmid was constructed by Genepharma (Shanghai, China). Packaging of AAV-shRNA-P2X4 was then performed. In brief, AAV-shRNA-P2X4 plasmid was transfected into HEK 293 cells (Manassas, VA, USA) using Lipofectamine 2000. Forty-eight hours later, the culture supernatants were collected and purified using Lenti-X™ Concentrator (Clontech, 631,257, Mountain View, USA).

### Hematoxylin–eosin (HE) staining

Brain tissues were collected and embedded in paraffin. The block was cut into 5-μm-thick slices. After being dewaxed and rehydrated, the sections were dyed with Ehematoxylin (Solarbio, BP-DL001, Beijing, China) for 5 min and stained with eosin (Solarbio, BP-DL001) for 3 min. The slices were observed under an optical microscope (Olympus, Tokyo, Japan).

### qRT-PCR

After 24 h of ICH, total RNA in mouse ipsilateral hemispheres was prepared using TRIZOL reagent (Thermo Fisher Scientific, 15596026CN). The RNAs were then synthesized into the first cDNAs by a SuperScript III First-Strand kit (Thermo Fisher Scientific A48571). Afterward, real-time PCR was performed with SYBR Green (Thermo Fisher Scientific, A46110). Different gene expressions were calculated using the 2^−∆∆Ct^ method and normalized to β-actin. Primer sequences used in the study were shown in Supplementary table 1.

### Western blot analysis

The ipsilateral brain tissues were removed 24 h after ICH and lysed in RIPA lysis buffer (Biocolors, 11,814,389,001, Shanghai, China). Protein extracts were electrophoretically resolved with SDS-PAGE and transferred to a polyvinylidene fluoride membrane (Roche, GVWP02500, Basel, Switzerland). After blockade with 5% non-fat milk, the blots were then incubated with primary antibody against NLRP1 (1:2000, ABF22, Sigma, Missouri, USA), ASC (1:2000, SAB4501315, Sigma), pro-caspase-1 (1:2000, PRS3459, Sigma), caspase-1 (1:2000, AB1871, Sigma), or β-actin (1:2000, A1978, Sigma) overnight at 4 °C, followed by peroxidase-conjugated secondary antibody (Cell Signaling Technology, MA, USA). The blots were visualized using an electrochemiluminescence detection kit (Amersham, Little Chalfont, UK).

### Enzyme-linked immunosorbent assay (ELISA)

After 24 h of ICH, approximately 600 μl of blood was collected from each mouse and centrifuged at 3,000 g for 10 min to separate serum. The serum levels of TNF-α, IL-6, and IL-1β were determined using corresponding ELISA kits according to the instructions of the manufacturer (R & D System, lot 318,592, Abingdon, UK).

### Statistical analysis

Data were presented as mean ± SD. The significance of differences between the two groups and more than two groups were identified by Student’s t-test and One-way ANOVA followed by Tukey’s test respectively. *P* < 0.05 was considered statistically significant.

### Ethics approval and consent to participate

All animal experiments were approved by the Institutional Animal Care and Use Committee of ShangRao People’s Hospital. And all methods are reported in accordance with ARRIVE guidelines. We confirm that all methods were performed in accordance with the relevant guidelines and regulations.

## Results

### P2X4 is highly expressed in the brain tissues of ICH mice

**Figure 1:**
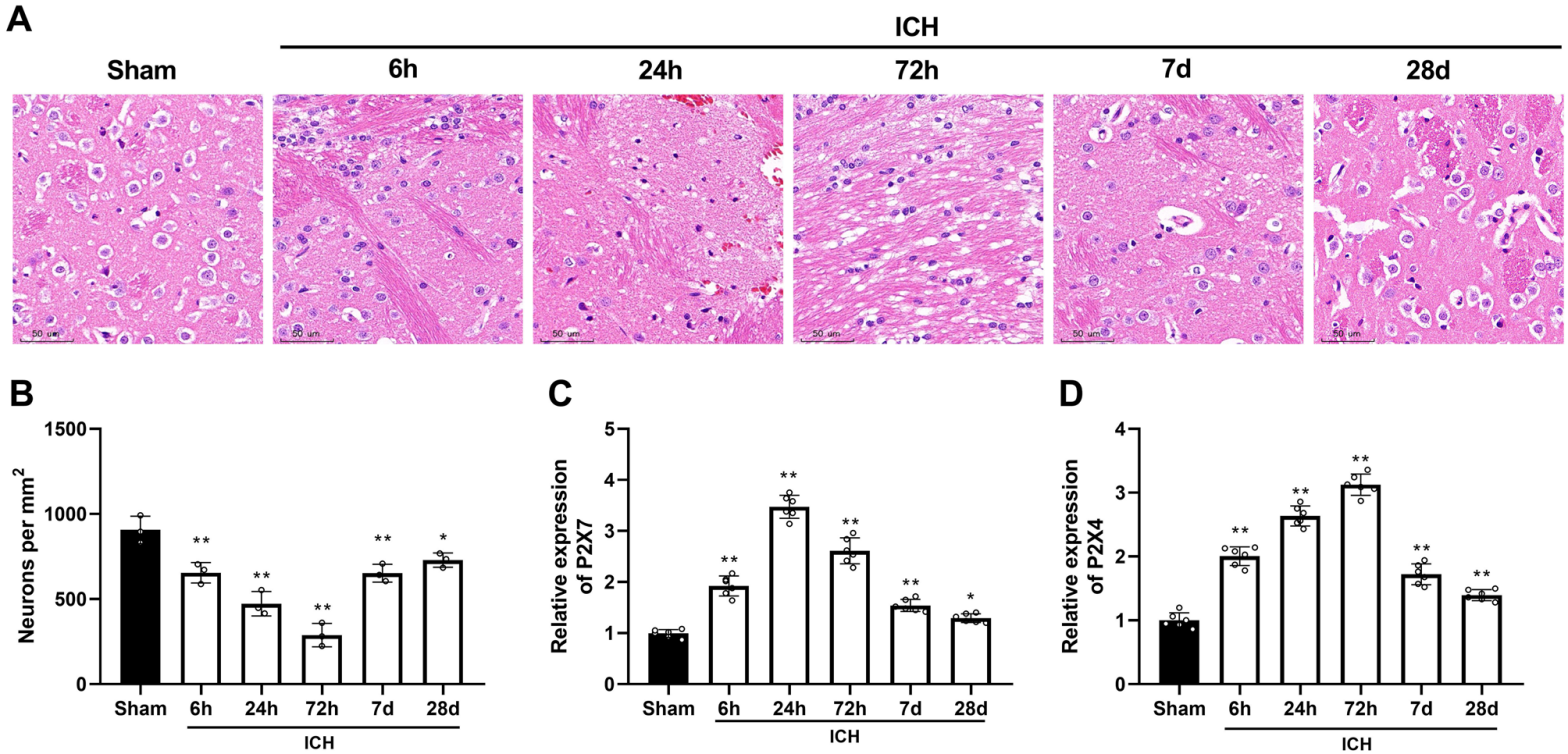
P2X4 is highly expressed in the brain tissues of ICH mice. (**A**) Hematoxylin–eosin (HE) staining of mouse brain tissues was performed in sham group, 6 h, 24 h, 72 h, and 7d-post ICH. Scale bar = 50 um (**B**) The number of surviving neuronal cells was quantified. (**C**) The mRNA level of P2X7 mouse ipsilateral hemispheres was detected by qRT-PCR at different time points. (**D**) The mRNA level of P2X4 mouse ipsilateral hemispheres was detected by qRT-PCR at different time points. One-Way ANOVA and Tukey’s multiple comparisons test was used. N = 6. ** *P* < 0.01 versus Sham.

H&E staining displayed histological changes in brain tissues caused by ICH. ICH led to severe inflammatory infiltration into brain tissues and gliocyte and capillary proliferation around hematoma. The most serious injury occurred 72 h-post ICH. The hematoma in brain tissues was alleviated 7 days after ICH operation (Fig. 1A). In addition, the number of neurons was significantly reduced in the ICH group compared to the sham group and reached its lowest value at 72 h after ICH and started to grow after 7 d (Fig. 1B). Then, P2X7 mRNA expression in the hemispheres after ICH was determined. Compared to sham-operated mice, P2X7 expression progressively increased over time in ICH mice, peaking at 24 h post-ICH (Fig. 1C). The results showed similar changes in mRNA expression of P2X7 and P2X4. Compared with sham-operated mice, P2X4 expression in ICH mice was elevated with increased time and was highest 72 h-post ICH. P2X4 expression on the 28th after ICH was decreased but still significantly higher than sham group (Fig. 1D). These results suggested that P2X receptor family played an important role in intracerebral hemorrhage.

### P2X4 knockdown alleviates ICH-induced short-term neurological deficits of mice

**Figure 2:**
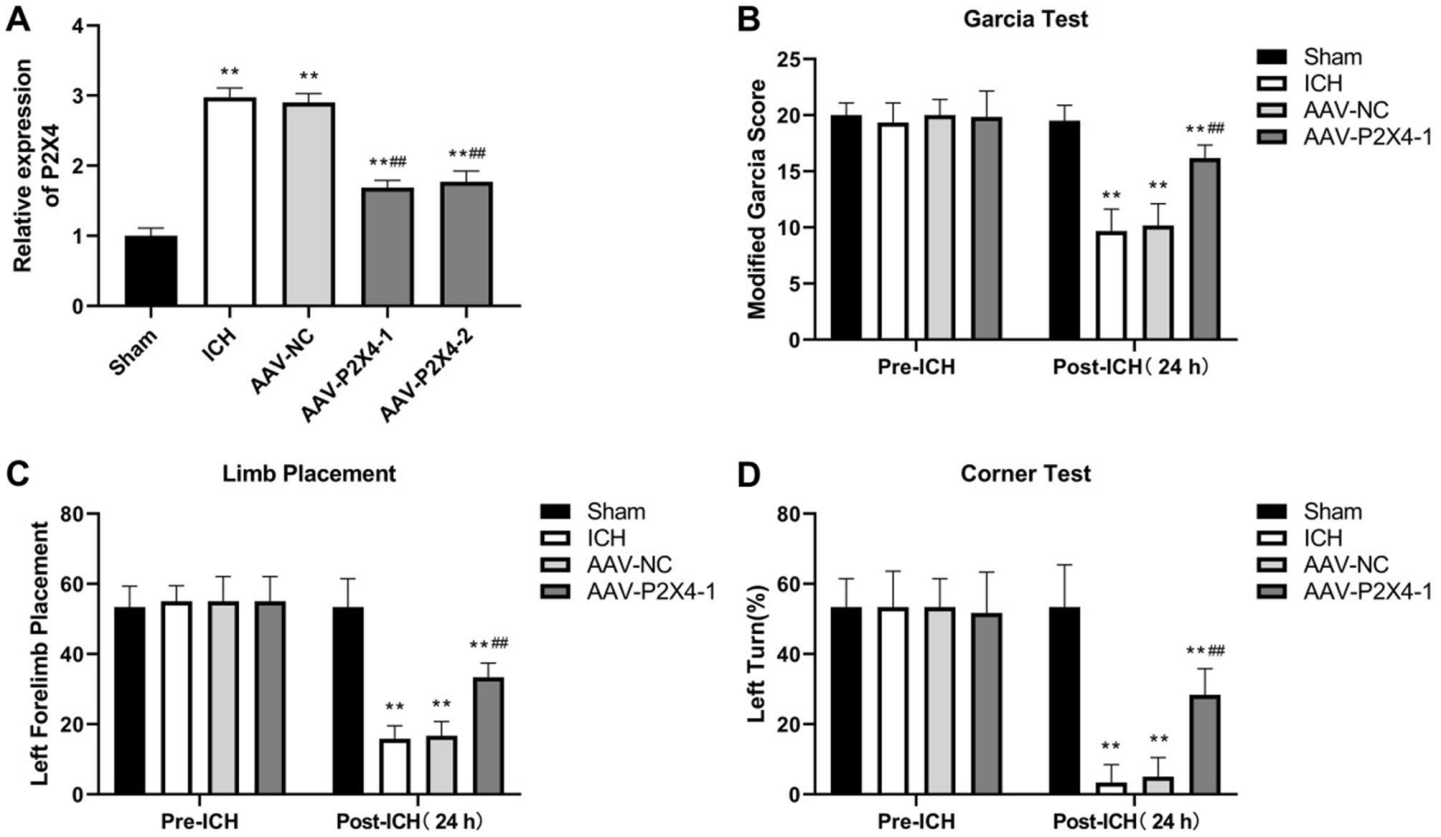
P2X4 knockdown alleviates ICH-induced short-term neurological deficits of mice. (**A**) P2X4 expression was detected by qRT-PCR 24 h-post ICH. (**B**–**D**) Garcia test (**B**), limb placement test (**C**), and corner turn test (**D**) were conducted before ICH and 24 h after ICH. One-Way ANOVA and Tukey’s multiple comparisons test was used. N = 6. ** *P* < 0.01 versus Sham, ## *P* < 0.01 versus ICH.

AAV-shP2X4 was injected to knock down P2X4 in the mouse brain. The efficiency of P2X4 knockdown in mice was detected using qRT-PCR. P2X4 mRNA expression increased by ICH was markedly reduced after infection with AAVes harboring two different shRNAs against P2X4. AAV-P2X4-1 showed higher knockdown efficiency and therefore AAV-P2X4-1 was selected for follow-up study. (Fig. 2A). Then, the short-term neurological deficits of mice were assessed both 4 h before ICH and 24 h after ICH. ICH mice showed statistically decreased performance in the Garcia test, limb placement test, and corner turn test 24 h after ICH compared to their performance before ICH. Infection of AAV-shP2X4 significantly improved the neurological functions of ICH mice (Fig. 2B–D), indicating that P2X4 knockdown alleviates ICH-induced short-term neurological deficits in mice.

### P2X4 silencing mitigates ICH-induced long-term neurological deficits of mice

**Figure 3:**
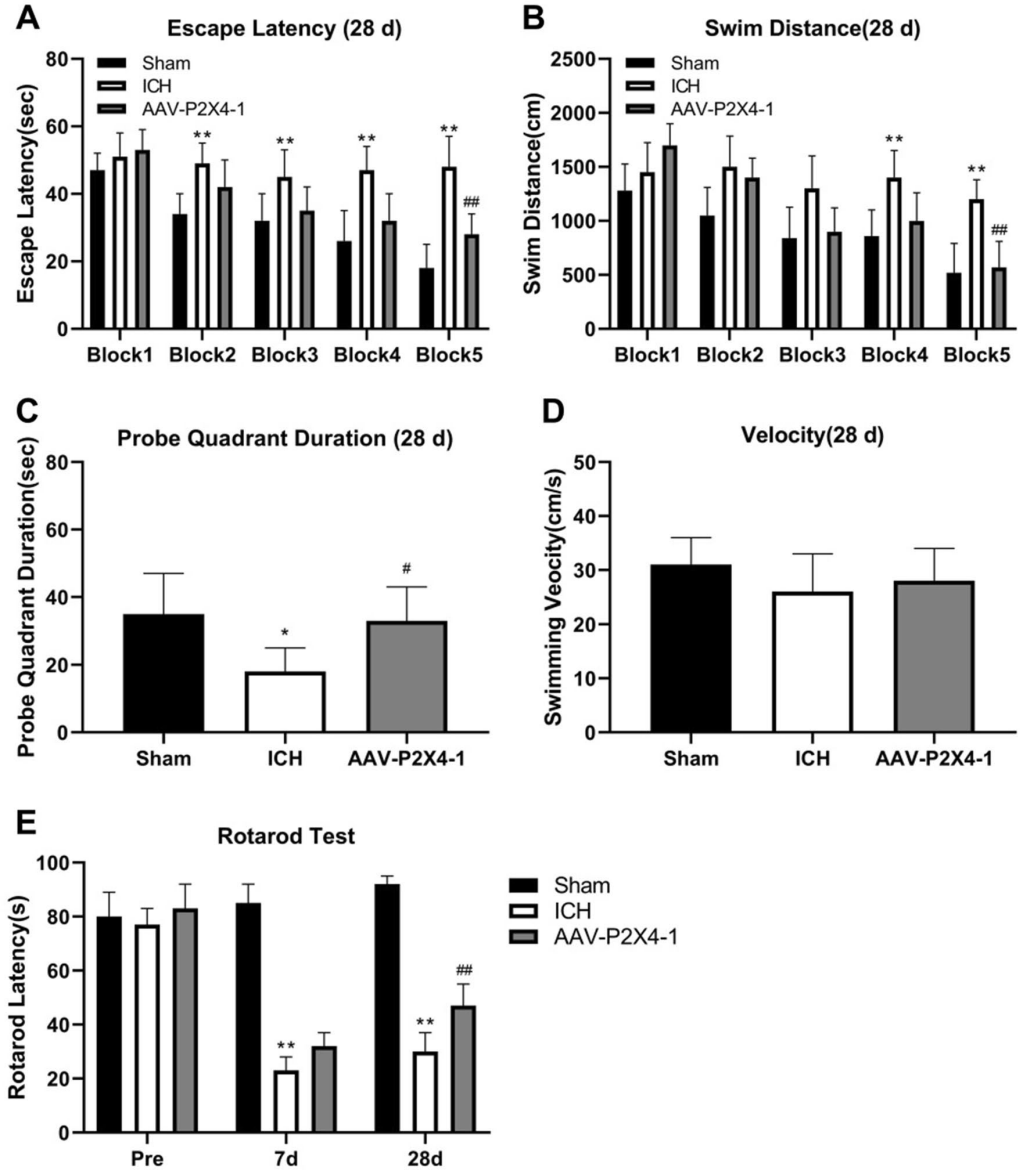
P2X4 silencing mitigates ICH-induced long-term neurological deficits of mice. (**A**–**D**) The escape latency (**A**), swimming distance (**B**), probe quadrant duration (**C**), and velocity (**D**) of mice were recorded at 28 d after ICH. (**E**) The rotarod test was conducted before ICH and 7 days and 28 days after the operation. One-Way ANOVA and Tukey’s multiple comparisons test was used. N = 6. ** *P* < 0.01, * *P* < 0.05 versus Sham, ## *P* < 0.01, # *P* < 0.05 versus ICH.

Morris water maze test and rotarod test were carried out 28 days after operation for the examination of ICH-induced long-term neurological impairment. The results in Fig. 3A and B showed that ICH mice exhibited the longer escape latency and swimming distance than sham-operated mice. The mice in AAV-shP2X4 group exhibited better performance in escape latency and swimming distance. The probe quadrant duration in ICH group was shorter than those in Sham group and then elevated by AAV-shP2X4 (Fig. 3C). However, the velocity of mice did not display a significant difference between these groups (Fig. 3D). The rotarod test was conducted before ICH, 7 days and 28 days after the operation. There was no change between the rotarod test of ICH group and that of AAV-shP2X4 7 days after the operation. However, P2X4 silencing improved mouse behaviors in the rotarod test 28 days after ICH (Fig. 3E).

### The expression and secretion of pro-inflammatory cytokines after ICH are repressed by P2X4 knockdown

**Figure 4:**
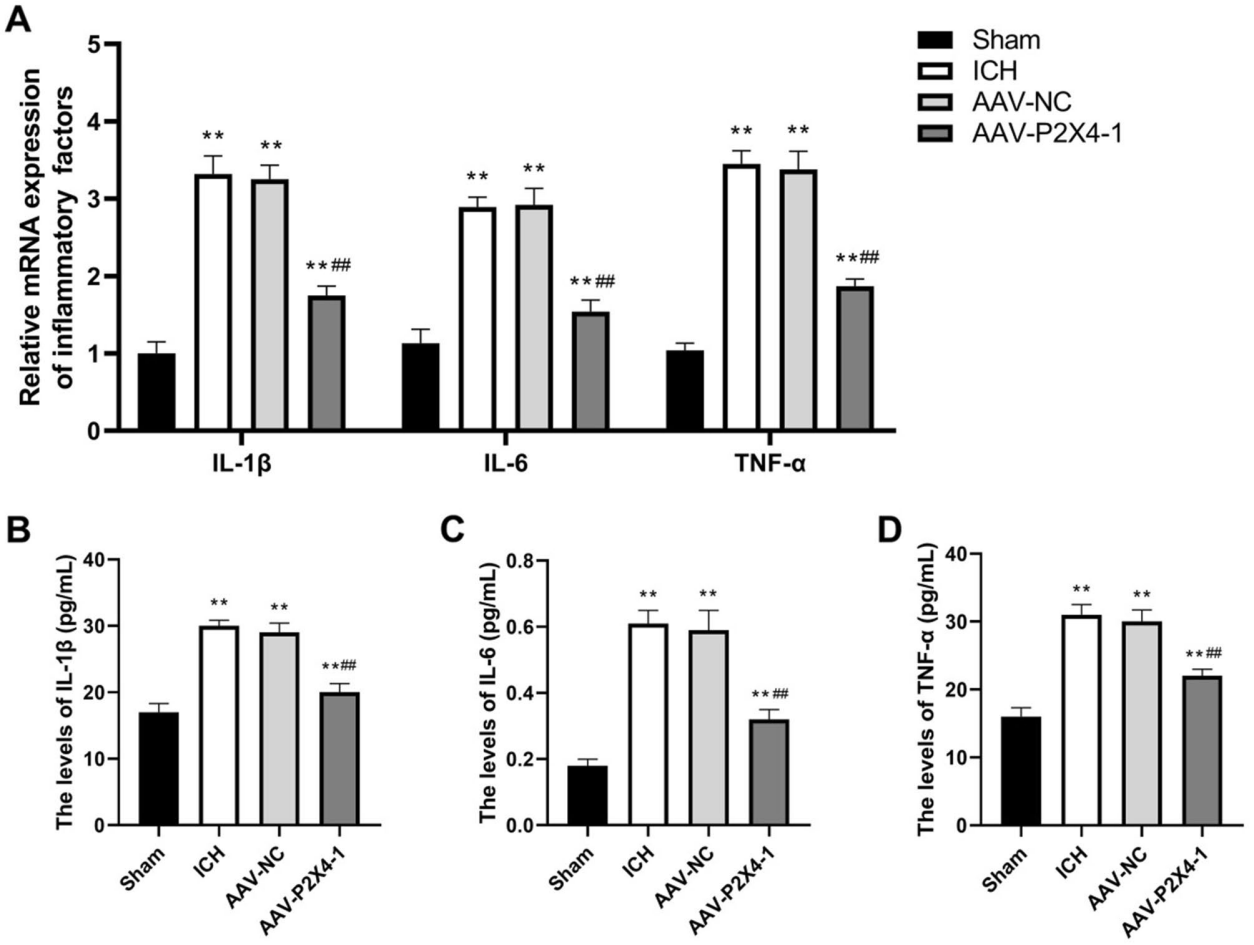
The expression and secretion of pro-inflammatory cytokines after ICH are repressed by P2X4 knockdown. (**A**) The tissue mRNA levels of IL-1β, IL-6, and TNF-α were determined with qRT-PCR. (**B**–**D**) The serum levels of IL-1β (**B**), IL-6 (**C**), and TNF-α (**D**) were detected with ELISA. One-Way ANOVA and Tukey’s multiple comparisons test was used. N = 6. ** *P* < 0.01 versus Sham, ## *P* < 0.01 versus AAV-NC.

The expression and secretion of pro-inflammatory cytokines including TNF-α, IL-6, and IL-1β were determined. The results in Fig. 4A showed that the mRNA levels TNF-α, IL-6, and IL-1β in mouse brain tissues were markedly increased upon ICH operation, and then were reversed by P2X4 inhibition. Similar alterations in the serum levels of these pro-inflammatory cytokines were also observed (Fig. 4B–D). The results above revealed that P2X4 knockdown restricted the expression and secretion of pro-inflammatory cytokines after ICH.

### P2X4 silencing blocks the NLRP1/Caspase-1 pathway in ICH

**Figure 5:**
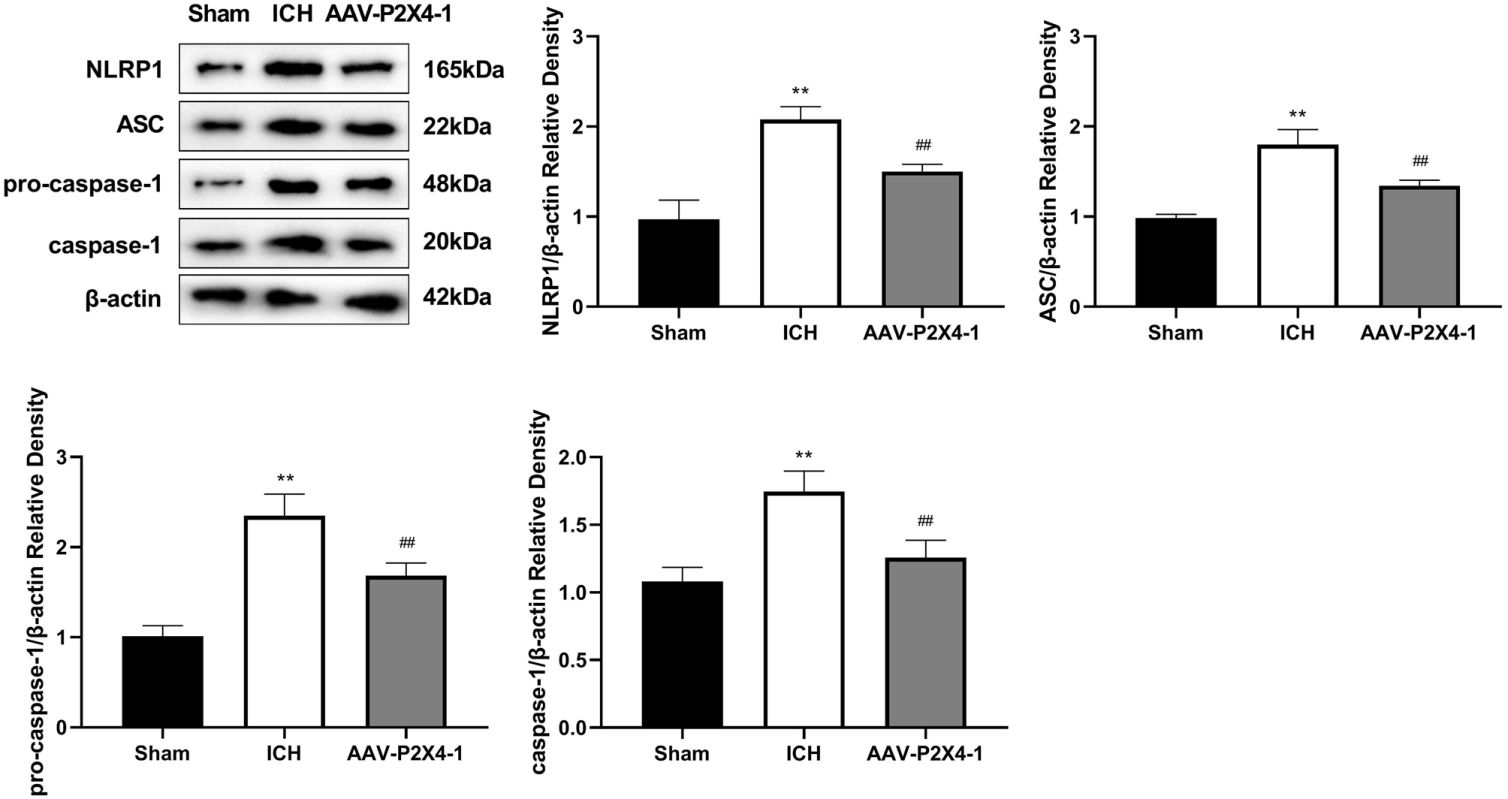
P2X4 inhibition blocks the NLRP1/Caspase-1 pathway in ICH. The protein levels of NLRP1, ASC, and pro-caspase-1 in mouse brain tissues were detected with Western blot. One-Way ANOVA and Tukey’s multiple comparisons test was used. N = 6. ** *P* < 0.01 versus Sham, ## *P* < 0.01 versus ICH.

We performed western blot analysis to determine the proteins involved in NLRP1/caspase-1 pathway. We found that ICH increased the protein levels of NLRP1, ASC, pro-caspase-1, and caspase-1 in mouse brain tissues, and P2X4 knockdown down-regulated ICH-induced expression of these proteins (Fig. 5).

### The NLRP1/Caspase-1 pathway mediates the effects of P2X4 silencing on neurological function and inflammation

**Figure 6:**
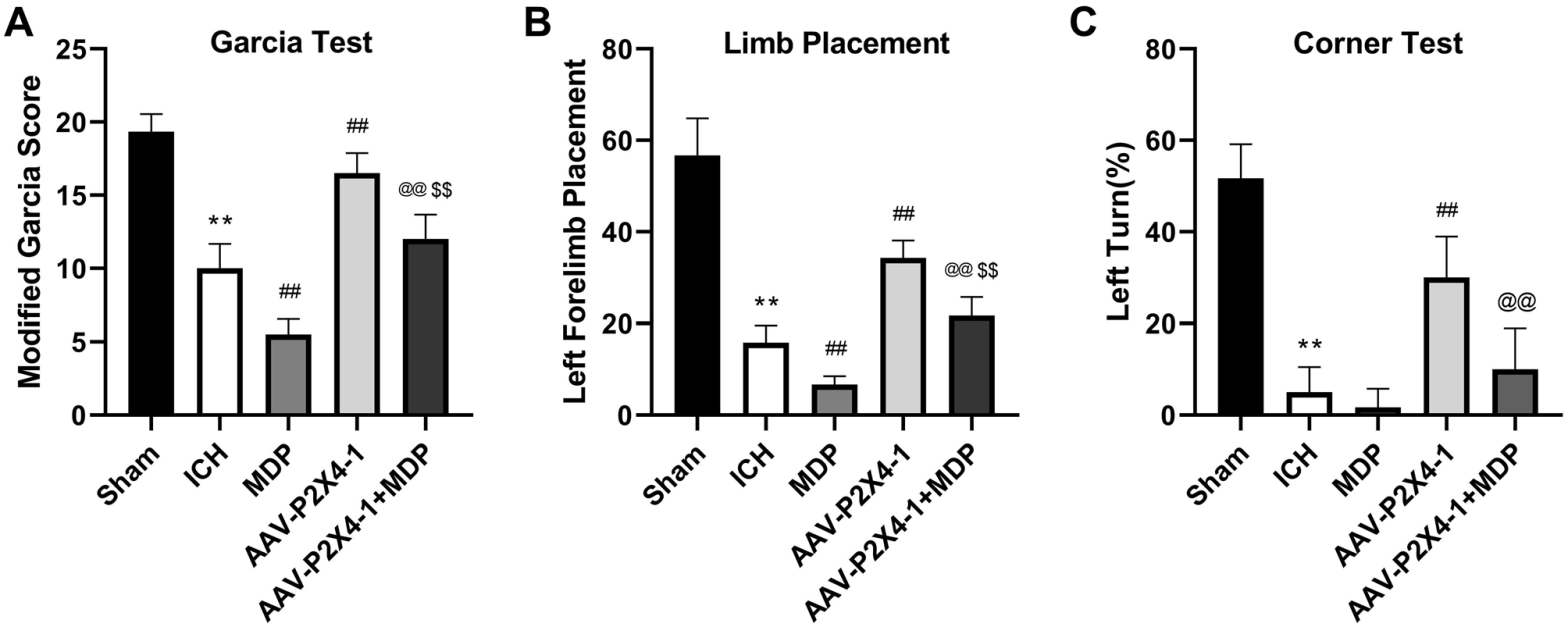
Muramyl dipeptide (MDP) aggravates neurological impairment which was mitigated by P2X4 silencing. (**A**–**C**) Garcia test (**A**), limb placement test (**B**), and corner turn test (**C**) were conducted 24 h after ICH. One-Way ANOVA and Tukey’s multiple comparisons test was used. N = 6. ** *P* < 0.01 versus Sham, ## *P* < 0.01 versus ICH, and @@ *P* < 0.01 versus AAV-P2X4-1.

**Figure 7:**
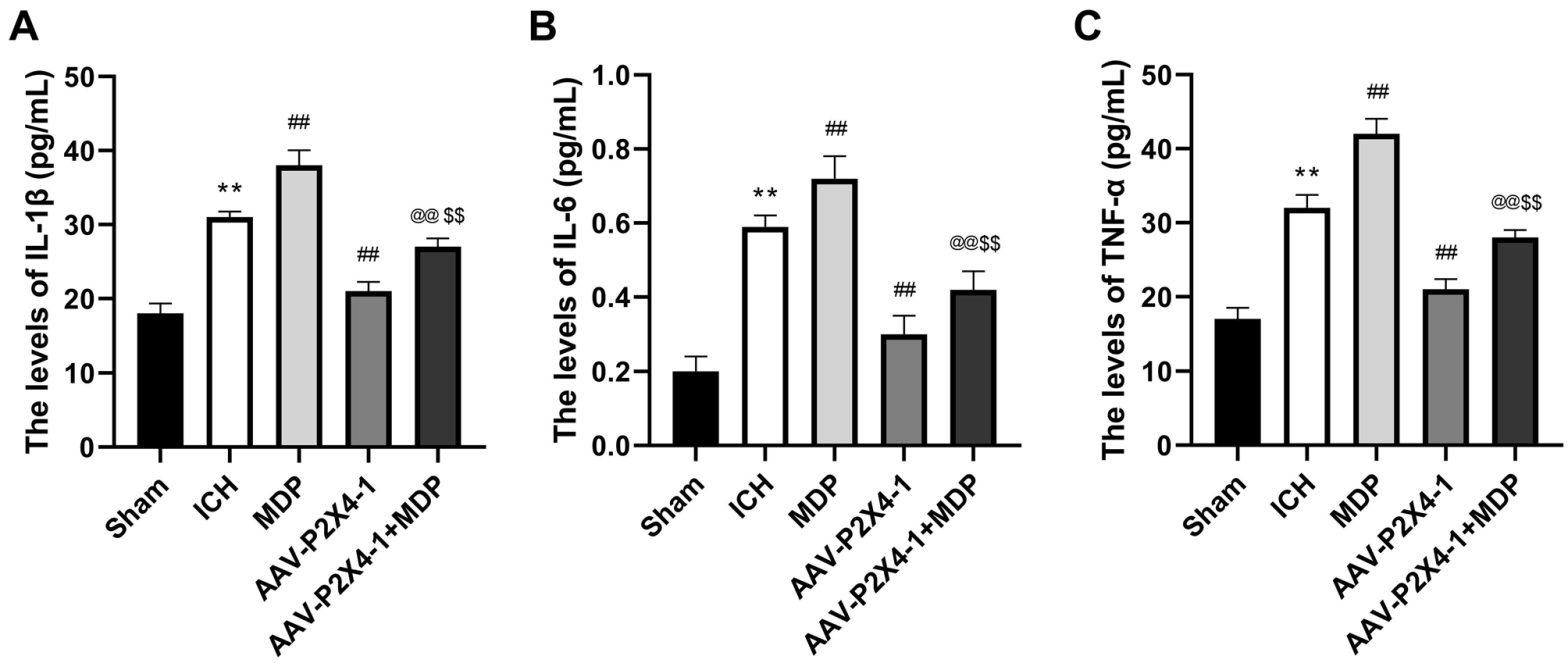
The NLRP1/Caspase-1 pathway mediates the effects of P2X4 silencing on neurological function and inflammation. (**A**–**C**) The serum levels of IL-1β (**A**), IL-6 (**B**), and TNF-α (**C**) were detected with ELISA. One-Way ANOVA and Tukey’s multiple comparisons test was used. N = 6. ** *P* < 0.01 versus Sham, ## *P* < 0.01 versus ICH, and @@ *P* < 0.01 versus AAV-P2X4-1.

To verify whether the NLRP1/Caspase-1 pathway was involved in the regulation of P2X4 in ICH, we treated ICH mice with NLRP1 activator MDP and then assessed the short-term neurological deficits of mice and inflammation in brain tissues. The results of Garcia test, limb placement, and corner test showed that MDP aggravated neurological impairment which was mitigated by P2X4 silencing (Fig. 6A–C). Besides, MDP also offset the inhibitory effects of P2X4 knockdown on TNF-α, IL-6, and IL-1β secretion (Fig. 7A–C). These results demonstrated that P2X4 regulation neurological function and inflammation by affecting the NLRP1/Caspase-1 pathway in a mouse ICH model.

## Discussion

The inflammation upon ICH would promote the formation of brain edema around hematoma, leading to a more severe and durable injury^25^. In this study, ICH induced P2X4 overexpression, neurological deficits, and neuroinflammation in mice. P2X4 silencing alleviated short- and long-term neurological impairment and reduced the expression and release of TNF-α, IL-6, and IL-1β. Additionally, NLRP1/Caspase-1 pathway was blocked by P2X4 knockdown, and NLPR1 activator abrogated the effect of P2X4 knockdown on neurological function and inflammation.

P2X4 is expressed in most cell types in the central and peripheral nervous systems^26^. Up-regulation of P2X4 is observed mainly in microglia but also in neurons and Schwann cells after nerve injury^27^. In the previous research, after nerve injury, P2X4 is up-regulated in spinal microglia at the transcriptional and translational levels due to several factors^28^. Besides, the expression of P2X4 in brain tissue is also up-regulated in a diabetic neuropathic pain model^29^. Primary brain injury after ICH is mainly caused by the compression and destruction of the adjacent tissue by hematoma formation, and the inflammation after ICH promotes the formation of brain edema around hematoma^30^. Herein, we found elevated P2X4 expression and hematoma formation in the mouse brain tissues in the ICH model. There are many neurologic manifestations of ICH, including motor dysfunction, sensory impairment, and cognitive impairment^31^. We assessed the neurological functions of ICH mice and found that P2X4 silencing alleviated ICH-induced short- and long-term neurological deficits, indicating the neuroprotective effect of P2X4 down-regulation in ICH.

P2X4 functions not only in central and peripheral nervous systems but also in other organic damages. P2X4 role in inflammation has been widely investigated in the brain or other organs/tissues. For instance, P2X4 is demonstrated to be required for the inflammation that contributes to both cerebral aneurysm formation and growth^32^. P2X4 suppression retards joint inflammation and damage in arthritis caused by collagen^33^. P2X4 receptor antagonists NC-2600 and NP-1815-PX exert anti-inflammatory effects in a murine colitis model^34^. The secondary brain injury following ICH involves multiple pathological and physiological factors, among which neuroinflammation induced by microglia/macrophage activation is of them^35^. Neuroinflammation is the main determinant of acute neuronal injury and neurodegeneration. In the rat brain trauma model, the P2X4 inhibition effectively reduces the inflammation and apoptosis of microglia and mitigates nervous system defects^36^. Moreover, P2X4 expression is increased after stroke, and its deletion is found to be neuroprotective^37^. Our research revealed that P2X4 down-regulation restricted the tissue expression and serum levels of TNF-α, IL-6, and IL-1β, thus alleviating neuroinflammation after ICH. This was similar to the findings that elevation of P2X4 expression caused by high-concentration free fatty acids (FFA) is associated with elevated mRNA expression and release of TNF-α in RAW264.7 macrophages, and FFA-induced inflammation may be reversed by the P2X4 antagonist^38^. Furthermore, P2X4 activation has been shown to increase inflammation^39^. Therefore, P2X4 may contribute to neuroinflammation upon ICH.

The classical inflammasome complex is composed of a cytosolic sensor NLR protein, an adaptor protein ASC, and an effector caspase pro-caspase-1^40^. Activation of the pro-inflammatory Caspase-1 protease results in the processing and maturation of IL-1β and IL-18^41^. P2X4 receptor forms a large conductance pore on the cell membrane, contributing to ion efflux and following inflammasome activation^42^. After spinal cord injury, spinal inflammasome signaling is impaired in P2X4R-deficient mice, leading to reduced IL-1β levels and infiltration of neutrophil- and monocyte-derived M1 macrophages^43^. The role of P2X4 in NLRP3 inflammasome has been reported before. For instance, P2X4 enhances the P2X7-dependent activation of inflammasomes, resulting in the increased release of IL-1β and IL-18^22^. Additionally, induction of kidney NLRP3 inflammasome signaling after renal ischemia and reperfusion was significantly attenuated in P2X4-deficient mice^44^. P2X4 inhibitor 5-BDBD significantly reduced the axotomy-dependent up-regulation of all three inflammasome-related genes NLRP3, caspase-1, and IL-1β^45^. Whereas, the function of P2X4 in NLRP1 inflammasome activation is little explored. MDP is a molecule derived from bacterial cell walls and acts as an agonist for the NOD2 (nucleotide-binding oligomerization domain-containing protein 2) receptor^46^. Studies have shown that MDP can be an activator of NLRP1 and NLRP3^47,48^. In the present study, P2X4 knockdown inhibited the expression of NLRP1, ASC, and pro-caspase-1 which was induced by ICH. To confirm that there is a correlation between P2X4 and NLRP1/Caspase-1 pathway, we treated the ICH mice with MDP after P2X4 knockdown. These results indicated that MDP offset the inhibitory effect of P2X4 suppression on neurological impairment and neuroinflammation after ICH. Hence, P2X4 may control the activation of NLRP1 inflammasome to regulate the neuroinflammation in ICH.

## Conclusion

7 days after ICH, the expression of P2X4 decreased, but remained higher than in the sham group. In the subsequent experiments, we found that in the case of decreased P2X4 levels after 28 days of ICH, the number of neurons recovered, but was still significantly lower than in the sham group. And compared to the sham group, the ICH mice had more pronounced neurological deficits and promoted pro-inflammatory cytokine expression, suggesting that although P2X4 expression was reduced, the mice did not fully recover from the disease. This also suggested that P2X4 might play an important role in intracerebral hemorrhage-induced neuroinflammation. Further studies showed that knockdown P2X4 further reduced short- and long-term neurological deficits and inflammation compared to the ICH group. In conclusion, ICH induces the up-regulation of P2X4 expression and neuroinflammation in the mouse. P2X4 inhibition alleviates ICH-induced neuroinflammation, and P2X4 may regulate neuroinflammation progression by affecting the NLRP1/caspase-1 pathway.

### Supplementary Information

Supplementary Table 1.

Supplementary Figures.

## Data Availability

The data that support the findings of this study are available from [ShangRao People’s Hospital] but restrictions apply to the availability of these data, which were used under license for the current study, and so are not publicly available. Data are however available from the authors upon reasonable request and with permission of [ShangRao People’s Hospital]. Yuanshui Wu should be contacted if someone wants to request the data from this study.
