# Supplementary material for: Purinergic neurotransmission receptor P2X4 silencing alleviates intracerebral hemorrhage-induced neuroinflammation by blocking the NLRP1/Caspase-1 pathway
Source: Sci Rep. 2023 Aug 31;13:14288. doi: 10.1038/s41598-023-40748-8 (PMC10471699; doi:10.1038/s41598-023-40748-8)
Supplement: Supplementary file 1 — Supplementary Table 1. [file 41598_2023_40748_MOESM1_ESM.docx]

Supplementary table 1 Primer sequences used in the study

| Primer name | Sequence (5'-3') |
| --- | --- |
| β-actin-F | CACCACAGCTGAGAGGGAA |
| β-actin-R | GGCCATCTCCTGCTCGAAG |
| P2X4-F | CTCATCCGCAGCCGTAAAGT |
| P2X4-R | AAAAGTTGGCGTTGGCGTAA |
| P2X7-F | TGTGTGCATTGACTTGCTCA |
| P2X7-R | CTTGCAGACTTTTCCCAAGC |
| IL-1β-F | AGAGCCCATCCTCTGTGACT |
| IL-1β-R | GCTTGGGATCCACACTCTCC |
| IL-6-F | CCAGTTGCCTTCTTGGGACT |
| IL-6-R | GTCTCCTCTCCGGACTTGTG |
| TNF-α-F | AGCCGATGGGTTGTACCTTG |
| TNF-α-R | ATAGCAAATCGGCTGACGGT |
