# Supplementary figures and images for: Purinergic neurotransmission receptor P2X4 silencing alleviates intracerebral hemorrhage-induced neuroinflammation by blocking the NLRP1/Caspase-1 pathway
Source: Sci Rep. 2023 Aug 31;13:14288. doi: 10.1038/s41598-023-40748-8 (PMC10471699; doi:10.1038/s41598-023-40748-8)

ASC

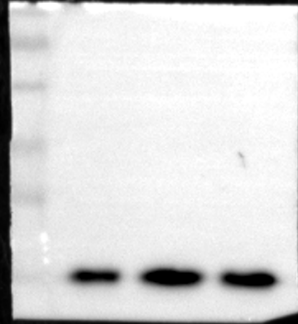

caspase-1

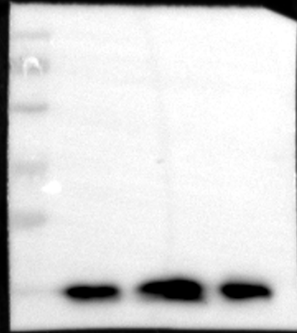

NLRP1

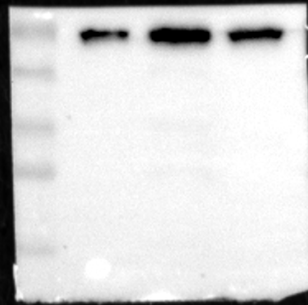

pro-caspase-1

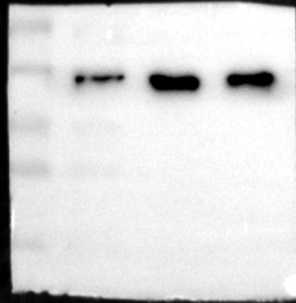

$\beta$ -Actin

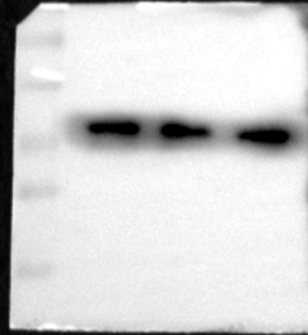

Supplement: Supplementary file 2 — Supplementary Figures. [file 41598_2023_40748_MOESM2_ESM.pdf]
